# Overhydration Is a Strong Predictor of Mortality in Peritoneal Dialysis Patients – Independently of Cardiac Failure

**DOI:** 10.1371/journal.pone.0158741

**Published:** 2016-07-14

**Authors:** Valérie Jotterand Drepper, Lars P. Kihm, Florian Kälble, Christian Diekmann, Joerg Seckinger, Claudia Sommerer, Martin Zeier, Vedat Schwenger

**Affiliations:** 1 Department of Nephrology, Geneva University Hospital, Geneva, Switzerland; 2 Department of Nephrology, University Hospital Heidelberg, Heidelberg, Germany; 3 Department of Nephrology, Zug Cantonal Hospital, Zug, Switzerland; 4 Department of Nephrology, Klinikum Stuttgart, Stuttgart, Germany; Robert Bosch Hospital, GERMANY

## Abstract

**Background:**

Overhydration is a common problem in peritoneal dialysis patients and has been shown to be associated with mortality. However, it still remains unclear whether overhydration per se is predictive of mortality or whether it is mainly a reflection of underlying comorbidities. The purpose of our study was to assess overhydration in peritoneal dialysis patients using bioimpedance spectroscopy and to investigate whether overhydration is an independent predictor of mortality.

**Methods:**

We analyzed and followed 54 peritoneal dialysis patients between June 2008 and December 2014. All patients underwent bioimpedance spectroscopy measurement once and were allocated to normohydrated and overhydrated groups. Overhydration was defined as an absolute overhydration/extracellular volume ratio > 15%. Simultaneously, clinical, echocardiographic and laboratory data were assessed. Heart failure was defined either on echocardiography, as a reduced left ventricular ejection fraction, or clinically according to the New York Heart Association functional classification. Patient survival was documented up until December 31^st^ 2014. Factors associated with mortality were identified and a multivariable Cox regression model was used to identify independent predictors of mortality.

**Results:**

Apart from higher daily peritoneal ultrafiltration rate and cumulative diuretic dose in overhydrated patients, there were no significant differences between the 2 groups, in particular with respect to gender, body mass index, comorbidity and cardiac medication. Mortality was higher in overhydrated than in euvolemic patients. In the univariate analysis, increased age, overhydration, low diastolic blood pressure, raised troponin and NTproBNP, hypoalbuminemia, heart failure but not CRP were predictive of mortality. After adjustment, only overhydration, increased age and low diastolic blood pressure remained statistically significant in the multivariate analysis.

**Conclusions:**

Overhydration remains an independent predictor of mortality even after adjustment for heart failure in peritoneal dialysis patients and should therefore be actively sought and managed in order to improve survival in this population.

## Introduction

Overhydration is a common problem in the dialysis population. In recent years much attention has thus been paid to the recognition of fluid overload and has led to the development of objective tools to assist clinical assessment. Bioimpedance spectroscopy offers the possibility of evaluating hydration status easily at the bedside [1] and has been validated for use in clinical practice [2–6].

In peritoneal dialysis (PD) patients, many studies have demonstrated an unexpectedly high rate of overhydration. Indeed, the EuroBCM study [7] showed that as many as 25% of PD patients are severely overhydrated. More recently, the IPOD-PD study [8] showed that less than half of patients are normohydrated (38.7%), whereas fluid overload >1.1 L occurs in 56.5%.

Overhydration and other factors including, amongst others, poor nutritional status [9], hypoalbuminemia [10], cardiac biomarkers such as N-terminal pro brain natriuretic peptide (NTproBNP) [11] and cardiac troponin (cTNT) [12,13], and elevated C-reactive protein (CRP) have been shown to correlate with mortality in both hemodialysis (HD) and PD patients [2,14,15]. However, it remains unclear whether overhydration per se is an independent predictor of mortality or whether it is mainly a reflection of underlying comorbidities such as cardiac dysfunction.

The purpose of our study was to assess hydration in peritoneal dialysis patients using bioimpedance spectroscopy and to investigate whether overhydration is an independent predictor of mortality.

## Materials and Methods

### Patients

Starting in June 2008, 54 peritoneal dialysis patients were analyzed and followed up to 6.5 years in this single centre, cross-sectional, non-interventional observational analysis. All PD patients over the age of 18 years were screened for eligibility. Patients with amputation of a major limb were excluded. Measurements were conducted once on each patient with the Body Composition Monitor (BCM) device (Fresenius Medical Care, Bad Homburg, Germany), and clinical, echocardiographic and laboratory data were collected simultaneously. Heart failure was defined either on echocardiography, as a reduced left ventricular ejection fraction, or clinically according to the New York Heart Association (NYHA) functional classification. Patient survival was documented up until the 31^st^ of December 2014 (up to 6.5 years follow-up). Death was regarded as an outcome event whilst patients who received a transplant, were switched to HD or left the study centre were censored at the time of the event. All patients were treated with low glucose degradation product solutions. All patients provided written informed consent and the study was approved by the ethics committee of the University Hospital Heidelberg (S-498). All procedures followed were in accordance with the Helsinki Declaration of 1975, as revised in 2000.

### Assessment of body composition and hydration status

Body composition was measured by multifrequency bioimpedance spectroscopy assessment using the BCM device, which has been validated by isotope dilution methods [16] and reference body-composition methods [17]. Extracellular water (ECW), intracellular water (ICW) and total body water (TBW) are determined from measured impedance data following the model of Moissl et al [4] using normal population data as reference. The measurement is performed by placing electrodes on one hand and one foot and entering current height and weight data into the machine. Bioimpedance is measured at 50 frequencies between 5 and 1000 kHz. Readings including the weight of the patient were performed with the peritoneal dialysate in situ.

Overhydration was defined according to the definition of Wizemann et al [2] and Devolder et al [6] as an absolute overhydration/extracellular volume (OH/ECW) ratio >0.15 (15%). Patients were then divided into 2 groups according to their results: normohydrated and overhydrated. Weight was assessed on a calibrated electronic weighing scale (Soehnle). Blood pressure was measured as the mean of three measurements in a sitting position using a standardized Omron electronic device (Omron M6; Omron Healthcare Europe, Milton Keynes, UK) with brachial cuff adjusted to the patient’s brachial circumference.

### Laboratory assessment

We assessed blood levels of CRP, the cardiac biomarkers troponin and NTproBNP, serum albumin concentration, haemoglobin and haematocrit on the day of BCM assessment.

### Statistical analysis

Statistical analysis was performed using SPSS 11·5 software (Chicago, IL USA). Data for continuous variables were expressed as means and SD. Categorical variables were expressed as absolute numbers and percentages. The T test for comparison of sample means between the 2 groups was used, the Fisher exact test for association between categorical variables and the Spearman’s linear correlation coefficient for correlation between continuous variables. Multivariable Cox regression models were applied using covariates with *P* < 0.1 in the univariate analysis, including heart failure and the cardiac biomarkers cTNT and NTproBNP. A Kaplan-Meier survival curve was generated and differences in survival were compared by the log-rank test. In this analysis patients who underwent kidney transplantation or were transferred to HD were censored at the time of transfer. The underlying assumptions of the proportional hazards model were tested and found to be valid. All p-values were two tailed. *P* < 0.05 was considered to be statistically significant.

## Results

### Patient characteristics

The characteristics of normohydrated and overhydrated patients are shown in Table 1.

**Table 1:** Patient characteristics.

	All (n = 54)	Normohydration (n = 38)	Overhydration (n = 16)	P-value
	Mean (SD)	Mean (SD)	Mean (SD)	T test
**Age (years)**	56.1 (15.5)	54.1 (15.8)	60.8 (14.1)	0.15
**Weight (kg)**	75.0 (12.4)	74.5 (12.9)	76.1 (11.4)	0.66
**BMI (kg/m2)**	25.5 (3.6)	25.6 (4.1)	25.3 (2.3)	0.79
**SBP (mmHg)**	123.0 (25.5)	121.8 (23.6)	125.8 (30.1)	0.60
**DBP (mmHg)**	76.2 (16.9)	76.6 (15.8)	75.1 (19.9)	0.76
**Diuresis (ml)**	1392 (932)	1496 (867)	1144 (1057)	0.21
**PUF (ml)**	714 (580)	599 (447)	989 (763)	0.02
**Furosemide (mg/day)**	284.6 (283.1)	214.0 (231.1)	452.5 (329.7)	0.004
**Spironolactone (mg/day)**	7.4 (14.3)	4.6 (11.4)	14.1 (18.2)	0.02
**Glucose load (g/day)**	160.8 (75.9)	150.0 (52.8)	185.2 (110.4)	0.12
**Time on PD (days)**	757 (869)	836 (885)	571 (825)	0.31
	N (%)	N (%)	N (%)	Fisher
**Male gender**	33 (61.1%)	22 (57.9%)	11 (68.8)	0.54
**CAD**	31 (51.9%)	20 (52.6%)	11 (68.8%)	0.37
**Diabetes**	17 (31.5%)	10 (26.3%)	7 (43.8%)	0.22
**Heart failure**	29 (53.7%)	17 (44.7%)	12 (75.0%)	0.07
**LVEF>55%**	27 (60.0%)	19 (61.2%)	8 (57.2%)	1
**LVEF 45–54%**	2 (4.4%)	1 (3.3%)	1 (7.1%)	0.53
**LVEF 30–44%**	4 (8.9%)	1 (3.3%)	3 (21.4%)	0.08
**LVEF<30%**	12 (26.7%)	10 (32.2%)	2 (14.3%)	0.28
**APD**	16 (29.6%)	12 (31.6%)	4 (25.0%)	0.75
**H transporter**	8 (16.0%)	3 (8.3%)	5 (35.8%)	0.03
**HA transporter**	27 (54.0%)	20 (55.6%)	7 (50.0%)	0.35
**LA transporter**	10 (20%)	9 (25%)	1 (7.1%)	0.25
**L transporter**	5 (10.0%)	4 (11.1%)	1 (7.1%)	1
**Extraneal use**	19 (35.2%)	12 (31.6%)	7 (43.8%)	0.53
**EPO**	28 (51.9%)	20 (52.0%)	8 (50.0%)	1
**ACEI/ARB**	38 (70.4%)	29 (76.3%)	9 (56.3%)	0.19
**Calcium antagonist**	22 (40.7%)	18 (47.4%)	4 (25.0%)	0.14
**ß-blocker**	45 (83.3%)	33 (86.8%)	12 (75.0%)	0.42
**Statins**	33 (61.0%)	24 (63.2%)	9 (56.3%)	0.76
**Transfer to HD**	23 (42.6%)	17 (44.7%)	6 (37.5%)	0.77

SBP, systolic blood pressure; DBP, diastolic blood pressure; PUF, peritoneal ultrafiltration; CAD, coronary artery disease; LVEF, left ventricular ejection fraction; APD, automated peritoneal dialysis; EPO, erythropoietin; ACE, angiotensin converting enzyme inhibitor; ARB, angiotensin receptor blocker.

Overhydration (OH/ECW ratio >15%) was found in 29.6% of PD patients. There were no significant differences between the 2 groups regarding gender, bodyweight, body mass index, known coronary heart disease and diabetes. There was also no significant difference in systolic or diastolic blood pressure between the 2 groups.

More than half of our patients (53,7%) were found to have heart failure NYHA 1 or more. 60% of all patients in our cohort had a left ventricular ejection fraction >55% with no significant difference between the euvolemic and overhydrated groups; however, data were missing in 9 patients. Overhydrated patients had significantly higher daily peritoneal ultrafiltration rates and cumulative diuretic dose; there was a trend towards less residual diuresis and more heart failure. There were also significantly more high transporter characteristics in the overhydrated group, although data were missing in 4 patients (2 in each group). Regarding PD prescription, there was no difference in Extraneal use and daily glucose load between the 2 groups. Use of antihypertensive drugs, EPO and statins was not significantly different between the 2 groups. Diabetes was present in 31.5% of all patients.

The mean overhydration volume obtained by BCM in overhydrated patients was 5.06L vs. 0.92L in normohydrated patients (*p*<0.001). The results of the bioimpedance spectroscopy for the 2 groups are shown in Table 2 below.

**Table 2:** Bioimpedance spectroscopy results.

	Normohydration (n = 38)	Overhydration (n = 16)	P-value
	Mean (SD)	Mean (SD)	T test
ICW (L)	19.30 (4.28)	18.90 (3.97)	0.73
ECW (L)	16.87 (2.93)	20.87 (4.03)	<0.001
TBW (L)	36.20 (6.86)	39.70 (7.33)	0.15
OH (L)	0.92 (1.14)	5.06 (2.64)	<0.001
FTM (kg)	25.10 (9.88)	23.30 (7.63)	0.27
LTM (kg)	39.10 (11.10)	38.60 (10.10)	0.87

ICW, intracellular water; ECW, extracellular water; TBW, total body water; OH, water in excess; FTM, fat total mass; LTM, lean total mass

Primary renal diseases were glomerulonephritis (24%), hypertensive nephrosclerosis (21%), diabetic nephropathy (20%), polycystic kidney disease (5%) and other non-specified renal diseases (30%).

### Laboratory findings

Overhydrated patients demonstrated significantly increased CRP, cardiac troponin (cTNT) and NTproBNP levels and significantly lower albumin concentration compared to normohydrated patients (Table 3). Hematocrit values did not differ significantly between the 2 groups.

**Table 3:** Laboratory data.

	Normohydration (n = 38)	Overhydration (n = 16)	P-value
	Mean (SD)	Mean (SD)	T test
**CRP (mg/L)**	5.3 (6.7)	10.4 (12.9)	0.02
**Albumin (g/L)**	39.2 (3.7)	35.2 (4.0)	0.001
**cTNT (mg/mL)**	0.047 (0.052)	0.098 (0.073)	0.006
**NTproBNP (pg/mL)**	10166 (18163)	26568 (33255)	0.023
**Hematocrit (%)**	31.0 (0.04)	33.0 (0.05)	0.21

### Survival

**Fig 1:**
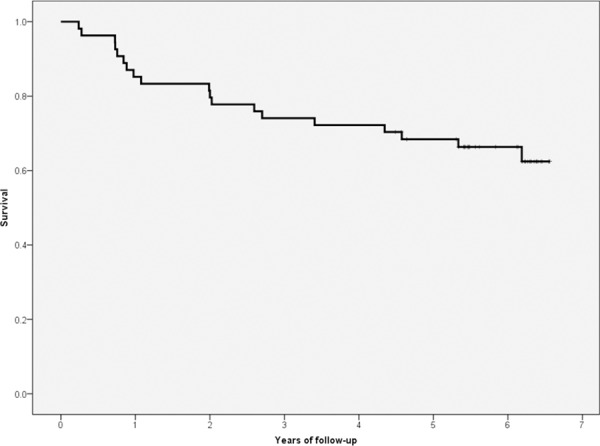
Overall survival.

**Fig 2:**
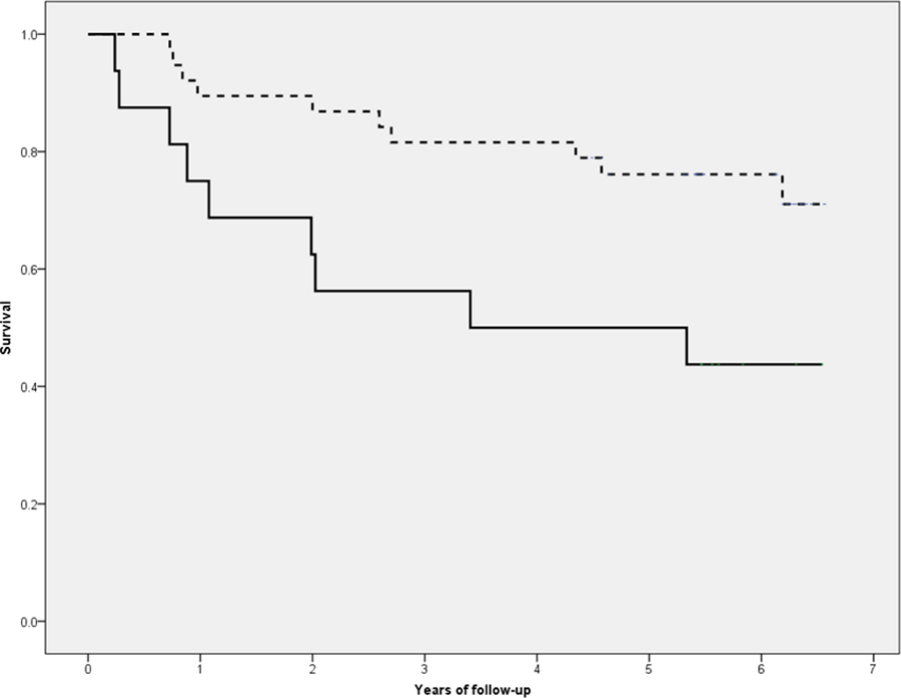
Survival curves in normohydrated and overhydrated patients. _ _ _ normohydrated group; ____ overhydrated group.

Nineteen patients (35.2%) died during follow-up (6.5 years). Survival probabilities were 85.2% at 1 year, 79.6% at 2 years, 74.1% at 3 years, 72.2% at 4 years, 68.4% at 5 years, and 66.3% at 6 years (Fig 1). Causes of death were: sudden cardiac death (5), myocardial infarction (4), sepsis unrelated to peritoneal dialysis (1) and stroke (1). In 8 patients the cause of death was unknown. Significantly more overhydrated patients died than euvolemic patients (Fig 2).

### Predictors of mortality

In the univariate analysis increased age, overhydration, low diastolic blood pressure, the cardiac biomarkers cTNT and NTproBNP, hypoalbuminemia (<36g/l), heart failure but not CRP were predictive of mortality. After adjustment only increased age, low diastolic blood pressure and overhydration remained statistically significant in the multivariate analysis as shown in Table 4.

**Table 4:** Predictors of mortality (Cox regression).

	Univariate associations		Multivariate model	
	Relative hazard (95% confidence interval)	P value	Relative hazard (95% confidence interval)	P value
**Overhydration, for 1 SD (0.11)**	2.19 (1.35–3.54)	0.001	7.82 (1.10–29.07)	0.002
**cTNT, for 1 SD (0.0634)**	2.30 (1.47–3.60)	<0.001	2.16 (0.86–5.39)	0.10
**NTproBNP, for 1 SD (24504)**	1.48 (1.13–1.95)	0.005	1.95 (0.93–3.96)	0.07
**CRP, for 1 SD (9.16)**	1.31 (0.90–1.89)	0.16	0.75 (0.26–2.15)	0.60
**Age (per year)**	1.08 (1.03–1.12)	0.001	1.16 (1.07–1.27)	0.008
**Heart failure (present vs absent)**	5.12 (1.48–17.75)	0.01	1.33 (0.22–8.22)	0.76
**Hypoalbuminemia (<36g/l)**	2.56 (1.01–6.50)	0.047	0.52 (0.07–3.63)	0.51
**DBP, for 1 SD decrease (16.9 mmHg)**	4.02 (1.97–8.19)	<0.001	8.10 (2.60–25.27)	<0.001

SD, standard deviation; DBP, diastolic blood pressure

**Fig 3:**
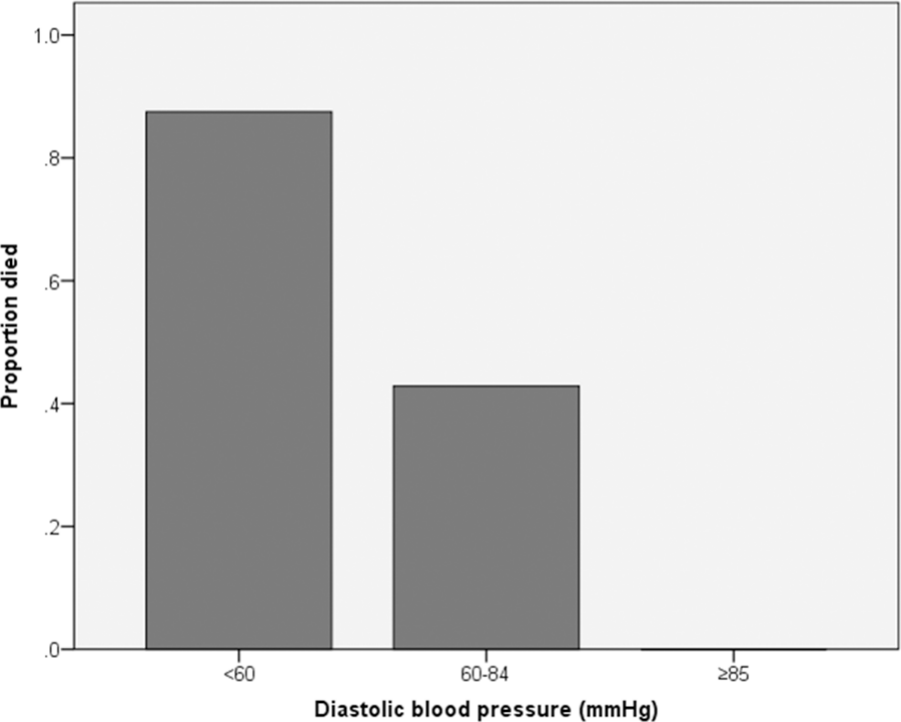
Correlation between diastolic blood pressure and death.

All 18 patients with a diastolic blood pressure >80mmHg survived, whereas 87,5% (7/8) patients with diastolic blood pressure <60mmHg died (Fig 3).

## Discussion

Mortality is high in PD patients. Our study shows a survival rate of only 66.3% at 6 years, which is similar to that found in a recent extensive retrospective American study [18]. In another recent Danish study, survival rate was even lower (40% at 6 years) [19]. It is therefore of utmost importance to identify factors influencing mortality, in particular those that are modifiable, in order to improve survival in this population.

Our study highlights the negative impact of overhydration on the survival of PD patients, overhydrated patients having a significantly higher mortality risk than euvolemic patients (2,2 x in the univariate analysis and up to 7,8 x in the multivariate analysis). There were no significant differences between normohydrated and overhydrated patients in terms of age, BMI, comorbidity, dialysis method and cardiac medication except for higher daily peritoneal ultrafiltration rate and cumulative diuretic dose in the overhydrated group, reinforcing the idea that overhydration per se is predictive of mortality. In the multivariate analysis overhydration still remained predictive of all-cause mortality after adjustment. In addition to classical predictive factors such as the cardiac biomarkers NTproBNP and troponin, inflammation markers (C-reactive protein) and hypoalbuminemia, we also took heart failure into account, which to our knowledge has not previously been done. This is highly relevant because overhydration may be falsely regarded as only a consequence of heart failure (HF), with attention essentially focused on treating the latter. We were able to demonstrate that heart failure is indeed a strong mortality predictor, but only in the univariate analysis (risk of dying 5 x higher in HF patients); it becomes statistically nonsignificant in the multivariate analysis. According to our results overhydration remains an independent mortality predictor even after adjustment for heart failure and should be per se a target of treatment, with euvolemia the goal.

We unexpectedly found that diastolic but not systolic blood pressure was strongly associated with mortality. Indeed, all 18 patients with a diastolic blood pressure >80mmHg survived whereas 87,5% patients with diastolic blood pressure <60mmHg died. After adjustment for comorbidity, lower diastolic blood pressure was also a strong independent predictor of mortality in the multivariate analysis. The prevalence of aortic insufficiency was 17% in the cohort as a whole with no significant difference between patients with lower (<60mmHg) and higher (>80mmHg) blood pressure. Among patients with lower blood pressure, only 1 was found to have aortic insufficiency, which suggests that low diastolic blood pressure was not due to this valvular disease in our cohort. In fact low diastolic blood pressure may reflect increased arterial stiffness as the underlying mechanism with subsequent increased cardiac afterload [20]. This association between low diastolic blood pressure and impaired survival has been recently described in a study evaluating a high coronary artery calcification score as predictor of all-cause mortality and cardiovascular outcome in PD patients, where patients with a high calcification score had lower diastolic blood pressure and a higher mortality risk [21]. A similar concept, known as reverse epidemiology of blood pressure, exists for systolic blood pressure: based on serial blood pressure measurements and echocardiographic findings Afshinnia et al [22] hypothesized that higher mortality associated with lower systolic blood pressure was in part mediated by worsening heart function in PD patients. The precise impact of low diastolic blood pressure on outcome in PD patients needs to be further investigated in dedicated studies.

In accordance with previous studies [23,24] the cardiac biomarkers cTNT and NTproBNP were predictive of mortality in PD patients in the univariate analysis. However biologic variation (i.e. the random fluctuation of a biomarker around a homeostatic set point) of NTproBNP seems to be large in the dialysis population as demonstrated in a very recent study [25] with concentrations needing to double or halve to confidently exclude change due to biologic variation alone. Large numerical changes in NTproBNP concentrations are potentially of no clinical significance and may result in false reassurance or alarm, which limits its usefulness in every day practice. Moreover, cardiac biomarkers were no longer associated with mortality after adjustment in our multivariate model, making them of lower interest.

We also found hypoalbuminemia to be predictive of mortality in the univariate analysis, an association previously described [10,26], but not in the multivariate model. This is in accordance with the results of Kang et al [27].

In contrast to other studies CRP was not associated with mortality in our study [28,29] although a systemic inflammatory state is believed to have a major impact on the survival of PD patients. However, as suggested by Jovanovic et al, CRP is probably not the best marker of inflammation. Without having tested other parameters like serum amyloid-A, as Jovanovic et al did [30], we cannot be sure that systemic inflammation would not have been significant.

Our study has several limitations: firstly, our collective is quite small, limiting the number of possible analyses (risk of model overfitting). In small collectives p-values may not show statistically significant differences which would have reached significance in larger ones.

Secondly, it is a cross-sectional study, and as such, no causal relationships can be assumed. Finally, the study was conducted in a single centre and may not be applicable to the general PD population.

## Conclusion

Identification of modifiable predictors of mortality in PD patients is of utmost importance in order to improve survival.

Overhydration is an independent predictor of mortality in this population even after adjustment for heart failure, and it should be actively sought and managed.

Low diastolic blood pressure is probably of importance in PD patients and its role in predicting mortality should be further investigated in dedicated prospective studies.
